# Duality of Tocopherol Isoforms and Novel Associations with Vitamins Involved in One-Carbon Metabolism: Results from an Elderly Sample of the LifeLines Cohort Study

**DOI:** 10.3390/nu12020580

**Published:** 2020-02-23

**Authors:** Camilo G. Sotomayor, Isidor Minović, Manfred L. Eggersdorfer, Ineke J. Riphagen, Martin H. de Borst, Louise H. Dekker, Ilja M. Nolte, Jan Frank, Sander K.R. van Zon, Sijmen A. Reijneveld, Jan C. van der Molen, Michel J. Vos, Jenny E. Kootstra-Ros, Ramón Rodrigo, Ido P. Kema, Gerjan J. Navis, Stephan J.L. Bakker

**Affiliations:** 1 Department of Internal Medicine, University Medical Center Groningen, University of Groningen, 9700 RB Groningen, The Netherlands; m.l.eggersdorfer@umcg.nl (M.L.E.); m.h.de.borst@umcg.nl (M.H.d.B.); l.h.dekker@umcg.nl (L.H.D.); g.j.navis@umcg.nl (G.J.N.);; 2 Department of Laboratory Medicine, University Medical Center Groningen, University of Groningen, 9700 RB Groningen, The Netherlands; i.minovic@umcg.nl (I.M.); i.j.riphagen@umcg.nl (I.J.R.); j.c.van.der.molen@umcg.nl (J.C.v.d.M.); m.j.vos01@umcg.nl (M.J.V.); j.e.kootstra@umcg.nl (J.E.K.-R.); i.p.kema@umcg.nl (I.P.K.); 3 Department of Epidemiology, University Medical Center Groningen, University of Groningen, 9700 RB Groningen, The Netherlands; i.m.nolte@umcg.nl; 4 Institute of Nutritional Sciences, University of Hohenheim, 70599 Stuttgart, Germany; jan.frank@nutres.de; 5 Department of Health Sciences, University Medical Center Groningen, University of Groningen, 9700 RB Groningen, The Netherlandss.a.reijneveld@umcg.nl (S.A.R.); 6 Molecular and Clinical Pharmacology Program, Institute of Biomedical Sciences, Faculty of Medicine, University of Chile, Santiago CP 8380453, Chile; rrodrigo@med.uchile.cl

**Keywords:** elderly, vitamin E, tocopherol, total lipids, insulin resistance syndrome, one-carbon metabolism, pyridoxal phosphate, cobalamin, folate, homocysteine

## Abstract

Whether the affinity of serum vitamin E with total lipids hampers the appropriate assessment of its association with age-related risk factors has not been investigated in epidemiological studies. We aimed to compare linear regression-derived coefficients of the association of non-indexed and total lipids-indexed vitamin E isoforms with clinical and laboratory characteristics pertaining to the lipid, metabolic syndrome, and one-carbon metabolism biological domains. We studied 1429 elderly subjects (non-vitamin supplement users, 60–75 years old, with low and high socioeconomic status) from the population-based LifeLines Cohort and Biobank Study. We found that the associations of tocopherol isoforms with lipids were inverted in total lipids-indexed analyses, which may be indicative of overcorrection. Irrespective of the methods of standardization, we consistently found positive associations of α-tocopherol with vitamins of the one-carbon metabolism pathway and inverse associations with characteristics related to glucose metabolism. The associations of γ-tocopherol were often opposite to those of α-tocopherol. These data suggest that tocopherol isoforms and one-carbon metabolism are related, with beneficial and adverse associations for α-tocopherol and γ-tocopherol, respectively. Whether tocopherol isoforms, or their interplay, truly affect the one-carbon metabolism pathway remains to be further studied.

## 1. Introduction

Vitamin E has been implicated in the prevention of age-dependent conditions such as type 2 diabetes and atherosclerosis [1,2,3,4]. Notably, these diseases are associated with relatively high lipid levels, which could hamper the assessment of their relationship with tocopherol isoforms. This is due to the fact that tocopherol isoforms are transported by lipoproteins, therefore often resulting in a direct association of tocopherol isoform levels with total lipids [5,6]. In both scientific and clinical practice, this has been long corrected for through calculation of the ratio of circulating concentrations of tocopherol isoforms over total fasting lipid concentrations (i.e., total cholesterol + triglycerides) [5,6]. However, because usually there is a direct association of total cholesterol with triglycerides, the aforementioned total lipids standardization method could lead to an unwanted double correction for variance that total cholesterol and triglycerides share, which could subsequently lead to unintended weakening—or even disappearance—and, in the worst case, inversion of otherwise existing direct associations. If this is true, one could anticipate that positive associations between tocopherol isoforms and lipids levels would turn into significantly inverse associations, rather than being absent, in total lipids-indexed analyses. 

From the aforementioned perspective, the components of the metabolic syndrome emerge as interesting biological characteristics to be—likewise—investigated, because it is established that they are associated with triglyceride levels [7,8,9,10,11]. Similarly, vitamins implicated in the one-carbon metabolism pathway (e.g., folate, cobalamin, and pyridoxal phosphate) are of particular interest in the elderly, because age-dependent conditions, including cardiovascular diseases and different kinds of cancer, may be partly explained by inadequate status of these vitamins [12]. Previous studies on tocopherol isoforms in elderly populations have mainly focused on potential associations with and effects on the immune system, infectious diseases, oxidative stress, and osteoporosis [13,14,15,16,17,18,19,20]. A study that investigated potential associations of circulating concentrations of both α- and γ-tocopherol in elderly subjects in the general population of Germany was more focused on diet and lipids and did not include components related to one-carbon metabolism [21].

We therefore set out to compare associations of non-indexed and total lipids-indexed concentrations of tocopherol isoforms in relation to characteristics pertaining to the lipid, metabolic syndrome, and one-carbon pathway biological domains in a large subset of elderly subjects (non-vitamin supplement users) from the LifeLines Cohort and Biobank Study.

## 2. Methods

### 2.1. Study Population

The LifeLines Cohort Study is an extensive cohort study based on the general population of the Northern part of the Netherlands, aiming to investigate the health of more than 152,000 subjects, who were enrolled via local general practices between 2006 and 2013 [22], as described in further detail elsewhere [23,24]. Study subjects could indicate whether family members were also interested in participating, and all interested subjects could register online. Excluding criteria were (i) deficient knowledge of the Dutch language, (ii) severe psychiatric or physical illness, and (iii) limited life expectancy (lower than 5 years). Study subjects completed questionnaires on diverse topics, e.g., general health, diet, physical activity, occurrence of diseases, medications, and personality traits. Study subjects were requested to attend the LifeLines Research locations for comprehensive health evaluation and to allow for storage of biological samples in the biobank underlying the LifeLines cohort study, including plasma, serum, and 24 h urine samples. None of the study subjects of the LifeLines Cohort Study received instructions concerning vitamin E intake or underwent specific standardization strategies of vitamin E intake, neither by means of medications nor through specific dietary counseling as a routine regimen. A written consent was signed by all study subjects at enrollment. The Medical Ethical Committee of the University Medical Center Groningen (UMCG, The Netherlands; METc 2007/152) approved the study, which was conducted in conformity with the Declaration of Helsinki.

### 2.2. Selection of Study Population

**Figure 1:**
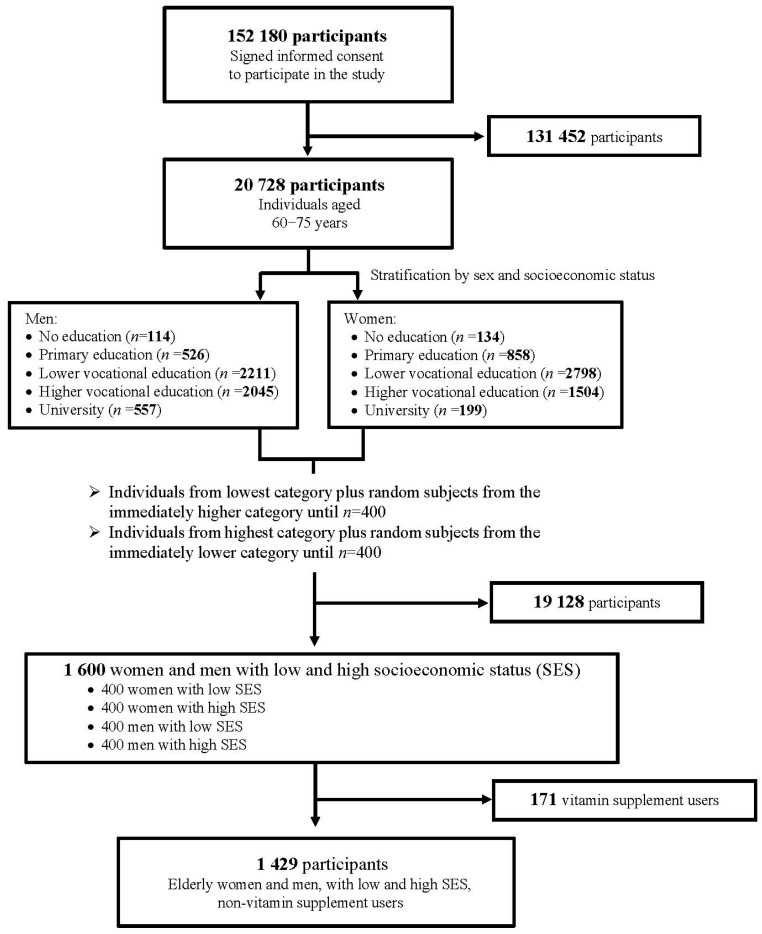
Flowchart of the 1429 study participants.

In total, 1600 subjects (aged between 60 and 75 years) were selected for the current study. This study population was composed of 400 women and 400 men belonging to low and high socioeconomic status. Vitamin supplements users were excluded, leaving 1429 study subjects, whose data are presented here (Figure 1).

### 2.3. Data Collection

Data collection on dietary intake (using a 110-item semiquantitative baseline food frequency questionnaire), education (using a self-administered questionnaire and categorization [25]), smoking habits, and other clinical assessments (height, weight, waist circumference, and blood pressure) are described in detail in Supplemental methods. 

### 2.4. Biochemical Measurements

To allow for the creation of a biobank and to allow for performance of an extensive laboratory investigation, venous blood samples were taken in the morning after an overnight period of 8 to 12 h of fasting and carried to the University Medical Center of Groningen, where the Central LifeLines Laboratory is located. We measured tocopherol isoforms by means of a validated method based on liquid chromatography tandem–mass spectrometry (LC–MS), as specified by Riphagen et al. [26]. Serum concentrations of low-density lipoprotein (LDL) cholesterol, high-density lipoprotein (HDL) cholesterol, total cholesterol, and triglycerides were measured by means of routine enzymatic assays based on spectrophotometric detection performed on a Roche Modular P chemistry analyzer (Roche, Basel, Switzerland). Fasting blood glucose was also assessed on this analyzer via the hexokinase method. HbA_1C_ and thyroid function tests were performed using a Cobas Integra 800 CTS analyzer (Roche Diagnostics Netherland BV, Almere, The Netherlands). Insulin resistance was calculated as the ratio of triglycerides over HDL cholesterol [27]. Creatinine and serum uric acid were assessed using an enzymatic colorimetric assay on a routing chemistry analysis platform (Roche Modular P, Roche, Basel, Switzerland). We measured high-sensitivity C reactive protein (hs-CRP) using nephelometry (BN II system Siemens, Marburg, Germany). Creatine was assessed by means of LC–MS [28]. Plasma pyridoxal phosphate was quantified as pyridoxal-5′-phosphate using a high-performance liquid chromatography method combined with fluorescence detection (FP-2020; Jasco Inc., Jasco, Easton, MD, USA) [29]. As detailed elsewhere, methylmalonic acid [30] and vitamin D3 [31] were assessed by means of LC–MS. Estimated glomerular filtration rate (eGFR) was calculated using an established formula (CKD-EPI [32]).

### 2.5. Statistical Analyses

Statistical analyses were performed using IBM SPSS Statistics v22.0 (IBM, Armonk, NY, USA). Baseline characteristics are summarized as mean (SD) for data with a normal distribution, median (interquartile range) for non-normally distributed data, or *n* (%) for categorical data. Serum vitamin E levels were indexed for total lipids by calculating their quotient over total lipids. We performed linear regression analyses with age and sex adjustment to evaluate the association of non-indexed and total lipids-indexed concentrations of tocopherol isoforms with clinical and laboratory characteristics. In sensitivity analyses, we also performed multivariate regression analyses with additional adjustment for body mass index (BMI). In all analyses, a 2-sided *p* < 0.05 was used as threshold for the determination of statistical significance.

## 3. Results

We included 727 men and 702 women with a mean (SD) age of 66 (4) years and a median (interquartile range) BMI of 26.4 (24.1–29.4) kg/m^2^. Serum α-tocopherol was 1.43 (1.25–1.64) mg/dL. Serum α-tocopherol (mg/dL)/total lipids (mg/dL) ratio × 1000 was 4.6 (0.7). Serum γ-tocopherol was 0.07 (0.05–0.09) mg/dL. Serum γ-tocopherol (mg/dL)/total lipids (mg/dL) ratio × 1000 was 0.21 (0.17–0.27); α-tocopherol and γ-tocopherol were positively associated (std. β = 0.35, *p* < 0.001).

### 3.1. Non-Indexed and Indexed α-Tocopherol and Biological Characteristics

Linear regression analyses of age- and sex-adjusted associations of non-indexed and total lipids-indexed α-tocopherol concentrations with clinical and laboratory characteristics are shown in Table 1. Among demographics, anthropometrics, lifestyle, and dietary intake, α-tocopherol was consistently associated with male gender. We observed that otherwise strongly positive associations of α-tocopherol with characteristics of the lipid domain became inverse, rather than absent, in total lipids-indexed analyses. In relation to glucose homeostasis, α-tocopherol was consistently inversely associated with fasting glucose and HbA_1C_. We also found that, irrespective of standardization, α-tocopherol was positively associated with pyridoxal phosphate, cobalamin, folate, and vitamin D3, whereas it was inversely associated with homocysteine. Finally, consistently inverse associations were also found between α-tocopherol and both free T3 and free T4.

### 3.2. Non-Indexed and Indexed γ-Tocopherol and Biological Characteristics

Linear regression analyses of age- and sex-adjusted associations of non-indexed and total lipids-indexed γ-tocopherol concentrations with clinical and laboratory characteristics are shown in Table 1. Among demographics, anthropometrics, lifestyle, and dietary intake, γ-tocopherol was consistently associated with BMI, smoking history, and alcohol intake, and inversely associated with age. We observed that otherwise strongly positive associations of γ-tocopherol with characteristics of the lipid domain became inverse, rather than absent, in total lipids-indexed analyses. Differently from α-tocopherol, in relation to glucose homeostasis, γ-tocopherol was consistently positively associated with fasting glucose and HbA_1C_. Likewise, we also observed that irrespective of standardization, γ-tocopherol was consistently negatively associated with pyridoxal phosphate, cobalamin, and folate. Finally, in relation to thyroid function, a consistent association was observed between γ-tocopherol and free T4.

### 3.3. Sensitivity Analyses

In sensitivity analyses with additional adjustment for BMI, associations of tocopherol isoforms with components of the metabolic syndrome were relatively less strong, whereas associations with other domains remained materially undisturbed (Supplemental Table S1).

## 4. Discussion

In elderly subjects of the general population, for the association of α- and γ-tocopherol with biological characteristics of the lipid domain, we found inversion of regression-derived coefficients from positive values in non-indexed analyses towards negative values in total lipids-indexed analyses, which may be indicative of overcorrection. Furthermore, irrespective of standardization, the most striking finding was the observation of consistently positive associations between α-tocopherol and vitamins involved in one-carbon metabolism. In contrast, despite a positive association between α- and γ-tocopherol themselves, γ-tocopherol was consistently inversely associated with the levels of vitamins involved in one-carbon metabolism, which may indicate a negative impact on the metabolic pathways associated with these vitamins. The associations of γ-tocopherol with characteristics related to glucose metabolism were also noteworthy, with direct associations of γ-tocopherol with BMI, fasting glucose, and HbA_1C_, which is consistent with the possibility that high intake of γ-tocopherol has adverse health effects, a finding which is in agreement with the observation that hepatic glucose and lipid metabolism are affected by tocopherol deficiency in rats and guinea pigs [33,34]. 

Similar to a recent publication on vitamin E and metabolic syndrome, we indexed our main analyses for total lipids [6]. Many reports, however, provide no indexation for circulating total lipids, which may certainly lead to findings that are actually driven by associations with circulating lipids rather than with circulating α-tocopherol [35]. Other reports have only indexed for total cholesterol or fasting triglycerides [21,36]. It should be realized that indexation by fasting triglycerides is likely inferior to that by total cholesterol exclusively, taking into account the much lower explained variance of α-tocopherol by fasting triglycerides than by total cholesterol [37] and that no indexation or indexation by only either total cholesterol or fasting triglycerides restraint the possibilities for a comparison of findings on associations and effects in the literature. We, therefore, provided analyses in which we performed no indexation and in which we indexed for total lipids. 

To the best of our knowledge, the current study is the first one to analyze potential associations of α-tocopherol with other vitamins and functional markers thereof. Noteworthy, multivitamin supplement users were excluded from the current analyses, which may support a rather causal nature of the associations of α-tocopherol with B vitamins and may suggest a potential role of α-tocopherol in the one-carbon metabolism pathway. Interestingly, despite a positive association between α- and γ-tocopherol themselves, most of the associations of γ-tocopherol with biological variables, if present, were opposite to those observed for α-tocopherol. It is well known that independent of its antioxidant properties, vitamin E is involved in cell signaling processes, and several genes have been shown to be differentially regulated by tocopherol isoforms [38,39]. Although the exact mechanisms that underlie tocopherol-dependent gene regulation remain to be elucidated, the most recent literature supports the potential of tocopherol isoforms to differentially regulate gene expression in several biological domains, including metabolism [40]. Although beyond the scope of the current study, Fischer et al. [40] recently reviewed gene regulatory activities of different tocopherol isoforms in relation to lipid uptake, cholesterol, steroid, and lipid metabolism, antioxidant defenses, inflammation, cell adhesion, immune response, cell signaling, cell cycle regulation, extracellular matrix, and cytoarchitecture. A growing body of evidence is in support of biological effects of tocopherol isoforms in directions that are consistent with beneficial health outcomes concerning α-tocopherol and adverse outcomes concerning γ-tocopherol, which is in line with the findings of the current study.

Duality of associations of α- and γ-tocopherol has been previously shown in relation to characteristics pertaining to the biological domains of inflammation, oxidative stress, vitamin D, anemia, mostly in the pulmonary domain, with physiological regulatory effects that are beneficial in the case of α-tocopherol and adverse for γ-tocopherol [37,41,42,43,44,45]. In agreement with those studies, in the current study we found that γ-tocopherol was directly associated with the expression of relevant components of the metabolic syndrome, e.g., BMI, fasting glucose, and HbA_1C_, which may be indicative of a true effect of γ-tocopherol on metabolism. The latter could suggest that γ-tocopherol has a detrimental biological impact either on the individual domains of the metabolic syndrome and one-carbon metabolism pathway or on both. 

The current study also reports intriguing observations of inverse associations between α- and γ-tocopherol and thyroid function (i.e., free triiodothyronine and thyroxine). We could find limited literature describing associations of α- or γ-tocopherol with free triiodothyronine and free thyroxine, yet a previous study of the effects of tocopherol isoforms on the chicken liver transcriptome provides hints about an effect on human biology [23]. Korošec et al. found that type 2 and type 3 5-deiodinase enzymes are liver genes remarkably affected by α- and γ-tocopherol, which may both link the levels of tocopherol isoforms to free triiodothyronine and thyroxine and provide a mechanistic link with components of the insulin resistance syndrome, with lipid homeostasis, and with one-carbon metabolism [33,34,45].

A major strength of our study is the large number of clinical and laboratory characteristics investigated in a large sample of elderly, non-vitamin supplements users, allowing the recognition of patterns rather than of associations with single biomarkers. It is also noteworthy that we investigated a study population composed of men and women with low and high socioeconomic status. Whereas, it should be realized that we reported on cross-sectional analyses, which precludes us from drawing hard conclusions about cause-and-effect associations. We also acknowledge as a limitation that we did not have access to data on erythrocyte α- and γ-tocopherol concentrations or on urinary metabolites of tocopherol isoforms, which could have given us further information on vitamin E status and circulating concentrations independent of lipids [37]. Finally, in the current study we did not investigate tocopherol isoforms other than those presented in the manuscript. On the basis of the current findings, it could be interesting to extend future studies with data on the other six naturally occurring vitamin E compounds (namely, β- and δ-tocopherol and α-, β-, γ-, and δ-tocotrienols) and to investigate potential associations with one-carbon metabolism and glucose-related components of the metabolic syndrome.

## 5. Conclusions

In the current study, we found interesting associations of tocopherol isoforms with B vitamins implicated in the trans-sulfuration and one-carbon metabolism pathways, as well as with characteristics related to glucose metabolism; it is noteworthy that these associations were in opposing directions for α- and γ-tocopherol isoforms. Remarkably, these opposite associations were found regardless of a direct association between the two isoforms. These data may suggest that these tocopherol isoforms or their interplay could have effects on human metabolism, possibly by influencing the liver transcriptome and that of other tissues such as the adipose tissue, which together could affect metabolism in directions that are in agreement with previous studies reporting either beneficial or adverse effects for α-tocopherol and γ-tocopherol, respectively.

## Figures and Tables

**Table 1:** Baseline characteristics of the study population and their associations with non-indexed and total lipids-indexed concentrations of tocopherol isoforms.

Baseline Characteristics	*n* = 1429	α-Tocopherol		γ-Tocopherol	
		Standardization		Standardization	
		None	Lipids	None	Lipids
**Demographics and Anthropometrics**					
Age, years ^†^	66 ± 4 ^§^	−0.04	−0.01	−0.08 **	−0.07 *
Sex, male ^†^	727 (51) ^¶^	0.17 ***	0.08 **	0.10 ***	−0.04
Body mass index, kg/m^2^	26.4 (24.1–29.4) ^¥^	−0.03	−0.22 ***	0.10 ***	0.06 *
Waist circumference, cm	101 ± 9	−0.02	−0.17 ***	0.08 **	0.04
Systolic blood pressure, mmHg	133 (122–145)	0.08 **	−0.11 ***	0.08 **	0.002
Diastolic blood pressure, mmHg	75 (69–81)	0.08 **	−0.06 *	0.05	−0.02
**Smoking status**					
Never smoker	484 (34)	—	—	—	—
Former smoker	759 (53)	0.02	0.02	0.07 *	0.06 *
Current smoker	173 (12)	−0.004	−0.09 ***	0.02	−0.03
**Dietary intake**					
Total protein, g/d	69 (57–82)	−0.03	0.07 *	−0.05	−0.01
Animal protein, g/d	41 (32–50)	−0.02	0.03	−0.04	−0.03
Vegetable protein, g/d	28 (22–34)	−0.02	0.10 **	−0.04	0.02
Total carbohydrates, g/d	202 ± 74	−0.04	0.05	−0.09 **	−0.05
Total fat, g/d	72 ± 31	−0.03	0.06 *	0.01	0.06
Alcohol intake, g/d	6.2 (0.8–16.4)	0.06 *	0.06	0.09 **	0.07 *
Energy intake, kCal/d	1849 ± 643	−0.03	0.07 *	−0.03	0.01
Use of multivitamins	0 (0)		—	—	—
**Vitamins**					
Urinary creatine/creatinine ratio × 1000	14 (9–53)	0.06 *	<0.001	0.05	0.03
Pyridoxal phosphate, nmol/L	51 (36–76)	0.20 ***	0.22 ***	−0.12 ***	−0.12 ***
Cobalamin, nmol/L	285 (221–354)	0.07 **	0.09 ***	−0.15 ***	−0.14 ***
Methylmalonic acid, nmol/L	170 (138–217)	−0.03	−0.01	0.04	0.05
Folate, nmol/L	15.8 (10.8–23.3)	0.08 **	0.08 **	−0.12 ***	−0.10 ***
Homocysteine, µmol/L	12 (11–16)	−0.07 *	−0.12 ***	0.08 **	0.05
Vitamin D3, nmol/L	62.0 (47.0–76.8)	0.09 **	0.16 ***	−0.05	−0.03
**Laboratory characteristics**					
*Lipids*					
Total cholesterol, mg/dL	209 ± 42	0.71 ***	−0.06 *	0.30 ***	−0.13 ***
HDL cholesterol, mg/dL	58 (46–70)	0.03	0.40 ***	−0.04	0.10 ***
Non-HDL cholesterol, mg/dL	150 ± 40	0.69 ***	−0.21 ***	0.32 ***	−0.16 ***
LDL cholesterol, mg/dL	135 (108–162)	0.62 ***	−0.09 ***	0.25 ***	−0.15 ***
Triglycerides, mg/dL	98 (74–133)	0.46 ***	−0.51 ***	0.29 ***	−0.18 ***
Total lipid, mg/dL	309 (269–360)	0.73 ***	−0.38 ***	0.38 ***	−0.19 ***
*Glucose homeostasis*					
Glucose, mmol/L	5.2 (4.8–5.7)	−0.06 *	−0.20 ***	0.09 ***	0.07 **
HbA_1C_, %	5.8 (5.6–6.0)	−0.05 *	−0.14 ***	0.09 ***	0.10 ***
*Thyroid function*					
TSH, mU/L	2.4 (1.6–3.4)	0.08	0.11 *	0.03	0.04
Free T3, pmol/L	5.0 ± 0.6	−0.13 *	−0.13 *	−0.11 *	−0.09
Free T4, pmol/L	16.0 ± 2.4	−0.13 *	−0.15 **	−0.15 **	−0.16 **
*Kidney function and inflammation*					
Creatinine, µmol/L	75 (66–85)	0.05	−0.06 *	0.04	−0.02
eGFR, mL/min/1.73 m^2^	91 (79–104)	−0.10	0.11 **	−0.01	0.05
Urinary albumin, mg/24 hrs	5.2 (3.0–9.5)	−0.02	−0.06	−0.01	0.02
Uric acid, mmol/L	0.32 ± 0.07	0.07	−0.21 ***	0.15 **	0.05
hs-CRP, mg/L	1.5 (0.7–2.9)	0.09 *	0.02	0.10 *	0.08
*Liver characteristics*					
ASAT, U/L	24 (21–28)	0.001	−0.03	0.02	−0.02
ALAT, U/L	21 (16–26)	−0.001	−0.15 ***	−0.01	−0.10 *
Alkaline phosphatase, U/L	65 (56–76)	−0.001	−0.11 *	0.02	−0.02
γ-Glutamyltransferase, U/L	23 (18–34)	0.08	−0.10 *	0.04	−0.05

* *p* < 0.05, ** *p* < 0.01, and *** *p* < 0.001. ^§^ Mean (standard deviation), ^¶^ *n* (%), ^¥^ median (interquartile range); all such values. Associations between baseline characteristics and plasma α- and γ-tocopherol concentrations were tested via multivariable age- and sex-adjusted linear regression analyses. Std. β coefficients represent the difference (in SD) in α- or γ-tocopherol per 1-SD increment in continuous characteristics or the difference (in SD) in α- or γ-tocopherol compared to the implied reference group for categorical characteristics. ^†^ Associations were adjusted for age or sex, where applicable. ALAT, alanine aminotransferase; ASAT, aspartate aminotransferase; eGFR, estimated glomerular filtration rate; HDL, high-density lipoprotein; hs-CRP, high-sensitivity C-reactive protein; LDL, low-density lipoprotein; TSH, thyroid-stimulating hormone.

## Supplementary Materials

The following are available online at https://www.mdpi.com/2072-6643/12/2/580/s1, Table S1: Baseline characteristics of the study population and their associations with non-indexed and total lipids-indexed concentrations of tocopherol isoforms.
