# Supplementary material for: Duality of Tocopherol Isoforms and Novel Associations with Vitamins Involved in One-Carbon Metabolism: Results from an Elderly Sample of the LifeLines Cohort Study
Source: Nutrients. 2020 Feb 23;12(2):580. doi: 10.3390/nu12020580 (PMC7071362; doi:10.3390/nu12020580)
Supplement: Supplementary file 1 [file nutrients-12-00580-s001.pdf]

nutrients

Article

Duality of Tocopherol Isoforms and Novel Associations with Vitamins Involved in One Carbon Metabolism: Results from an Elderly Sample of the LifeLines Cohort Study

Camilo G. Sotomayor [1,*], Isidor Minović [2], Manfred L. Eggersdorfer [1], Ineke J. Riphagen [2], Martin H. de Borst [1], Louise H. Dekker [1], Ilja M. Nolte [3], Jan Frank [4], Sander K.R. van Zon [5], Sijmen A. Reijneveld [5], Jan C. van der Molen [2], Michel J. Vos [2], Jenny E. Kootstra-Ros [2], Ramón Rodrigo [6], Ido P. Kema [2], Gerjan J. Navis [1] and Stephan J.L. Bakker [1]

1 Department of Internal Medicine, University Medical Center Groningen, University of Groningen, 9700 RB Groningen, The Netherlands; m.l.eggersdorfer@umcg.nl (M.L.E.); m.h.de.borst (M.H.d.B.); l.h.dekker@umcg.nl (L.H.D.); g.j.navis@umcg.nl (G.J.N.); s.j.l.bakker@umcg.nl (S.J.L.B.)
2 Department of Laboratory Medicine, University Medical Center Groningen, University of Groningen, 9700 RB Groningen, The Netherlands; i.minovic@umcg.nl (I.M.); i.j.riphagen@umcg.nl (I.J.R.); j.c.van.der.molen@umcg.nl (J.C.v.d.M.); m.j.vos01@umcg.nl (M.V.); j.e.kootstra@umcg.nl (J.E.K.); i.p.kema@umcg.nl (I.P.K.)
3 Department of Epidemiology, University Medical Center Groningen, University of Groningen, 9700 RB Groningen, The Netherlands; i.m.nolte@umcg.nl
4 Institute of Nutritional Sciences, University of Hohenheim, Germany; jan.frank@nutres.de
5 Department of Health Sciences, University Medical Center Groningen, University of Groningen, 9700 RB Groningen, The Netherlands; s.k.r.van.zon@umcg.nl (S.K.R.v.Z.); s.a.reijneveld@umcg.nl (S.A.R.)
6 Molecular and Clinical Pharmacology Program, Institute of Biomedical Sciences, Faculty of Medicine, University of Chile, CP 8380453 Santiago, Chile; rrodrigo@med.uchile.cl
* Correspondence: c.g.sotomayor.campos@umcg.nl; Tel.: +31-050-361-0881

Supplemental Methods

Dietary intake

To assess dietary intake and use of vitamin supplements in the Lifelines Cohort, a 110-item semiquantitative baseline food frequency questionnaire (FFQ) assessing consumed food items over the previous month was developed and validated by the Wageningen University using the Dutch FFQTOOL™, in which food items were selected based on the Dutch National Food Consumption Survey of 1997/1998. Seven answer categories were used to assess consumption frequency, ranging from "not this month" to "6–7 days a week". Portion size was estimated by fixed portion sizes (e.g., slices of bread, pieces of fruit) and commonly used household measures (e.g., cups, spoons). Energy and macronutrient intakes were estimated from the FFQ data by using the Dutch food composition database of 2011 (NEVO).

Data on Education and Smoking Habits

Information about education and smoking was collected from a self-administered questionnaire. Educational level was categorized into four groups: (1) never been to school or elementary school only, (2) lower vocational or secondary schooling, (3) intermediate vocational schooling or intermediate/higher secondary schooling, or (4) higher vocational schooling or university. Since education is more differentiating than income in the egalitarian Dutch population, classification of socioeconomic status was based on educational status and defined as low (never been to school or elementary school only or completed lower vocational or secondary schooling) and high (completed higher vocational schooling or education) [Vart P et al. Clin J Am Soc Nephrol ***2015***, 10, 562–570]. Additionally, subjects were classified according to their smoking habits (non-smokers, former smokers, or current smokers).

Clinical Measurements

Anthropometric measurements (weight, height, and waist circumference) and blood pressure were measured by trained staff. The anthropometric measurements were performed without shoes. Body weight was measured to the nearest 0.1 kg. Height and waist circumferences were measured to the nearest 0.5 cm. Height was measured with a stadiometer placing heels against the rod and the head in Frankfurt plane position. Waist circumference was measured in standing position with a tape measure all around the body, at the level midway between the lower rib margin and the iliac crest. Systolic and diastolic blood pressures were measured 10 times during a period of 10 min, using an automated Dinamap Monitor (GE Healthcare, Freiburg, Germany). The average of the final three readings was used for each blood pressure parameter.

Table S1. Baseline characteristics of the overall study population, and their associations with unstandardized and quotient-standardized concentrations of plasma tocopherol species

			α-tocopherol Standardization		γ-tocopherol Standardization	
	n=1429		None	Quotient	None	Quotient
**Demographics and anthropometrics**						
Age, years, mean (SD)†	66	(4)	−0.04	−0.01	−0.09**	−0.07**
Gender, male, *n* (%)†	727	(51)	0.17***	0.08**	0.11***	0.04
Body mass index, kg/m², median (IQR)	26.4	(24.1–29.4)	−0.03	−0.22***	0.04	0.51*
Waist circumference, cms, mean (SD)	101	(9)	0.01	0.01	−0.003	−0.003
Systolic blood pressure, mmHg, median (IQR)	133	(122–145)	0.09**	−0.06**	0.06*	−0.01
Diastolic blood pressure, mmHg, median (IQR)	75	(69–81)	0.09*	−0.04	0.04	−0.02
Smoking status						
Never smoker, *n* (%)	484	(34)	—	—	—	—
Former smoker, *n* (%)	759	(53)	0.02	0.04	0.06*	0.06*
Current smoker, *n* (%)	173	(12)	−0.01	−0.11***	0.03	−0.02
**Dietary intake**						
Total protein, g/d, median (IQR)	69	(57–82)	−0.03	0.07*	−0.05	−0.01
Animal protein, g/d, median (IQR)	41	(32–50)	−0.02	0.04	−0.05	−0.03
Vegetable protein, g/d, median (IQR)	28	(22–34)	−0.03	0.08**	−0.03	0.02
Total carbohydrates, g/d, mean (SD)	202	(74)	−0.05	0.03	−0.08*	−0.05
Total fat, g/d, mean (SD)	72	(31)	−0.03	0.06	0.01	0.06
Alcohol intake, g/d, median (IQR)	6.2	(0.8–16.4)	0.06*	0.04	0.10**	0.07*
Energy intake, kCal/d, mean (SD)	1849	(643)	−0.03	0.06	−0.03	0.01
Use of multivitamins, *n* (%)	0	(0)	—	—	—	—
**Vitamins**						
Urinary creatine/creatinine ratio ×1000, median (IQR)	14	(9–53)	0.07*	0.03	0.04	0.02
Vitamin B6, nmol/L, median (IQR)	51	(36–76)	0.20***	0.20***	−0.11***	−0.11***
Vitamin B12, nmol/L, median (IQR)	285	(221–354)	0.07**	0.09**	−0.15***	−0.14***
Methylmalonic acid, nmol/L, median (IQR)	170	(138–217)	−0.03	−0.01	0.04	0.05
Folic acid, nmol/L, median (IQR)	15.8	(10.8–23.3)	0.08**	0.14***	−0.11***	−0.09***
Homocysteine, μmol/L, median (IQR)	12	(11–16)	−0.07*	−0.11***	0.08**	0.05
Vitamin D3, nmol/L, median (IQR)	62.0	(47.0–76.8)	0.08**	0.14***	−0.04	−0.03
**Laboratory parameters**						
Lipids						
Total cholesterol, mg/dL, mean (SD)	209	(42)	0.72***	−0.10***	0.33***	−0.12***
HDL cholesterol, mg/dL, median (IQR)	58	(46–70)	0.01	0.36***	0.01	0.14***
Non-HDL cholesterol, mg/dL, mean (SD)	150	(40)	0.69***	−0.21***	0.32***	−0.16***
LDL cholesterol, mg/dL, median (IQR)	135	(108–162)	0.63***	−0.11***	0.27***	−0.14***
Triglycerides, mg/dL, median (IQR)	98	(74–133)	0.52***	−0.49***	0.28***	−0.22***
Total lipid, mg/dL, median (IQR)	309	(269–360)	0.74***	−0.36***	0.38***	−0.20***
Glucose homeostasis						
Glucose, mmol/L, median (IQR)	5.2	(4.8–5.7)	−0.06*	−0.12***	0.05	0.05
HbA_{1C}, %, median (IQR)	5.8	(5.6–6.0)	−0.05	−0.08**	0.06*	0.09**
Thyroid function						
TSH, mU/L, median (IQR)	2.4	(1.6–3.4)	0.09	0.09	0.05	0.04
Free T3, pmol/L, mean (SD)	5.0	(0.6)	−0.13*	−0.13*	−0.11*	−0.09
Free T4, pmol/L, mean (SD)	16.0	(2.4)	−0.13*	−0.14**	−0.16**	−0.16**
Kidney function and inflammation						
Creatinine, μmol/L, median (IQR)	75	(66–85)	0.06	−0.04	0.03	−0.02
eGFR, mL/min/1.73 m², median (IQR)	91	(79–104)	−0.01	0.08	0.01	0.06
Urinary albumin, mg/24 hrs, median (IQR)	5.2	(3.0–9.5)	−0.02	−0.04	−0.02	−0.03
Uric acid, mmol/L, mean (SD)	0.32	(0.07)	0.08	−0.18***	0.13*	0.02

hs-CRP, mg/L, median (IQR)	1.5	(0.7–2.9)	0.09*	0.07	0.08	0.06
Liver parameters						
ASAT, U/L, median (IQR)	24	(21–28)	<0.001	–0.03	0.02	–0.03
ALAT, U/L, median (IQR)	21	(16–26)	–0.003	–0.13**	–0.03	–0.12*
Alkaline phosphatase, U/L, median (IQR)	65	(56–76)	–0.001	–0.10*	0.01	–0.02
γ-Glutamyltransferase , U/L, median (IQR)	23	(18–34)	0.08	–0.09	0.03	–0.07

**P*<0.05, ***P*<0.01, and ****P*<0.001. Associations between baseline characteristics and plasma α- and γ-tocopherol concentrations were tested via multivariable age-, sex- and body mas index-adjusted linear regression analyses. Std. β coefficients represent the difference (in SD) in α- or γ-tocopherol per 1-SD increment in continuous characteristics or for categorical characteristics the difference (in SD) in α- or γ-tocopherol compared to the implied reference group. †Associations were adjusted for age or gender, where applicable. hs-CRP, high-sensitivity C-reactive protein; eGFR, estimated glomerular filtration rate.
